# Controlled Defects of Fluorine-incorporated ZnO Nanorods for Photovoltaic Enhancement

**DOI:** 10.1038/srep32645

**Published:** 2016-09-02

**Authors:** Hock Beng Lee, Riski Titian Ginting, Sin Tee Tan, Chun Hui Tan, Abdelelah Alshanableh, Hind Fadhil Oleiwi, Chi Chin Yap, Mohd Hafizuddin Hj. Jumali, Muhammad Yahaya

**Affiliations:** 1 School of Applied Physics, Faculty of Science and Technology, Universiti Kebangsaan Malaysia, UKM Bangi, 43600 Selangor, Malaysia; 2 Department of Flexible and Printable Electronics, Chonbuk National University, Jeonju 561-756, Republic of Korea

## Abstract

Anion passivation effect on metal-oxide nano-architecture offers a highly controllable platform for improving charge selectivity and extraction, with direct relevance to their implementation in hybrid solar cells. In current work, we demonstrated the incorporation of fluorine (F) as an anion dopant to address the defect-rich nature of ZnO nanorods (ZNR) and improve the feasibility of its role as electron acceptor. The detailed morphology evolution and defect engineering on ZNR were studied as a function of F-doping concentration (*x*). Specifically, the rod-shaped arrays of ZnO were transformed into taper-shaped arrays at high *x*. A hypsochromic shift was observed in optical energy band gap due to the Burstein-Moss effect. A substantial suppression on intrinsic defects in ZnO lattice directly epitomized the novel role of fluorine as an oxygen defect quencher. The results show that 10-FZNR/P3HT device exhibited two-fold higher power conversion efficiency than the pristine ZNR/P3HT device, primarily due to the reduced Schottky defects and charge transfer barrier. Essentially, the reported findings yielded insights on the functions of fluorine on (i) surface –OH passivation, (ii) oxygen vacancies (V_o_) occupation and (iii) lattice oxygen substitution, thereby enhancing the photo-physical processes, carrier mobility and concentration of FZNR based device.

Chemical doping, defect-engineering, surface structure passivation and modification have been extensively practiced on semiconductor based nanomaterials to tailor their physical properties, electronic band structure, charge selectivity and optical behavior for photovoltaic performance enhancement. In the field of organic photovoltaics, a great research endeavor has been committed to replace fullerene with transparent metal-oxides (TMO) as electron acceptor in bilayer hybrid solar cells (HSC), in order to overcome the uncontrollable phase separation, fabrication complexity and air-stability issue of fullerene based device^1^,^2^. Solution-processed ZnO nanorods (ZNR), in particular, has been the most prominent TMO based inorganic electron acceptor latterly owing to its flexible synthesis route, high electron affinity and tunable morphology including size, diameter, density and rod-to-rod spacing to yield high surface-to-volume ratio^3^,^4^. In addition, the high hydrophobicity and vertical alignment of ZNR could facilitate the infiltration of polymer, thus providing a large donor-acceptor interfacial area and direct electron conduction pathway in vertically-stacked device^3^,^5^.

Nevertheless, the photovoltaic performance of ZNR as electron acceptors remains inferior to that of PCBM in HSC application primarily due to the abundancy of native defects. Therefore, the material properties and device performance of ZNR typically require further optimization such as doping, an approach commonly known for its efficacies and feasibility. Our previous studies have exemplified that the incorporation of metal dopants namely Bi, Ga, and Mg into ZNR could effectively address its defect-rich nature, which predominantly determines the photovoltaic performance of ZNR based device^6^,^7^,^8^. A key distinction of degenerately doped ZNR is that its material properties and electronic band structures are highly dependent on low energy native defects including V_o_, Zn interstitials (Zn_i_) and free hydroxyls (–OH) group^4^,^5^,^9^. Moreover, the photovoltaic performance and the charge transport of ZNR based device is also frequently hindered by the existence of electron and hole trap sites due to V_o_ and surface –OH group, respectively. This issue has limited the practical application of ZNR in organic photovoltaics. In this regard, a precisely controlled chemical doping could be the pragmatic approach in dictating the photovoltaic performance of ZNR based device.

Most of the literature concerning chemical doping on ZNR focused heavily on using metal-cation based dopants (Group I-III elements). On the contrary, the utilization of anion based dopants remains eluded in organic photovoltaic application. Generally, the incorporation of metal dopants into ZnO nanostructures suffers from a few shortcomings including (i) severe lattice distortion which disrupts the periodic network of ZnO matrix and overall charge transport efficiency, (ii) out-diffusion of the electrically active metal dopants during fabrication and (iii) less competency in eliminating the oxygen defects of ZnO^10^,^11^,^12^. To overcome these issues, we utilized a halogen group element, namely fluorine (F) simultaneously as an anion dopant and simultaneously, an oxygen defect quencher for ZNR in current work. The highly compatible ionic radius of F^−^ (1.36 Å) with O^2−^ (1.40 Å) as well as its electrically inactive nature as a dopant imply that F^−^ could be easily incorporated into ZnO matrix to enhance the electron acceptor properties of ZNR^10^. A recent report by Choi *et al*. confirmed that the incorporation of F into ZnO film could effectively suppress the O-related defects particularly V_o_ and surface hydroxyl group, thereby giving rise to the unorthodox optical behavior and electronic properties of ZnO film^12^. This phenomenon also implies the F doping is capable of eliminating the defect-induced charge trapping sites in ZNR. Therefore, it is of paramount importance to understand the defect-passivation mechanism of F dopant on the hierarchical ZNR arrays and the subsequent effects on its device performance.

In this report, we have successfully illustrated the incorporation pathway of F^−^ into ZnO host lattice network. By governing the interactions between F dopants and the oxygen defect states, the charge trapping sites in ZNR could be suppressed, thereby increasing the charge selectivity and extraction of ZNR to achieve better device performance. Our findings also reveal that the incorporation pathway of F dopants relies heavily on the concentration of NH_4_F (doping precursor), whereby it commenced by either forming dative hydrogen bond with surface –OH molecules or occupying the existing oxygen vacancies (V_o_) and progressively substituting the lattice oxygen. Fascinatingly, the incorporation of F^−^ into ZnO matrix resulted in the generation of extra delocalized electrons and phenomenal suppression of oxygen-related defects. As a consequence, the electron acceptor properties of ZNR were substantially enhanced and to be specific, the optimum device (10-FZNR) exhibited a two-fold higher power conversion efficiency (PCE) than that of pristine ZNR device. Prominently, the enhanced device performance can be attributed to larger interfacial area for exciton dissociation, higher carrier mobility and concentration and indefinitely, lesser defect-assisted recombination during charge transport by virtue of F-passivation effect. Furthermore, the crystallinity degradation and oxygen defects suppression phenomenon was also observed in 12-FZNR, exemplifying the degenerate semiconductor behavior which deteriorated its device performance. Comprehensively, this report presents novel findings and provide useful insights concerning anion-controlled passivation mechanism and the direct influence on charge transfer process at metal-oxide/polymer photovoltaic interface. Essentially, the peculiar tunable material properties of solution-processed FZNR such as low defects and excellent charge selectivity have accounted for its commendable role as TMO based electron collection layer for the future development of high performance organic and perovskite solar cells.

## Result and Discussions

**Figure 1:**
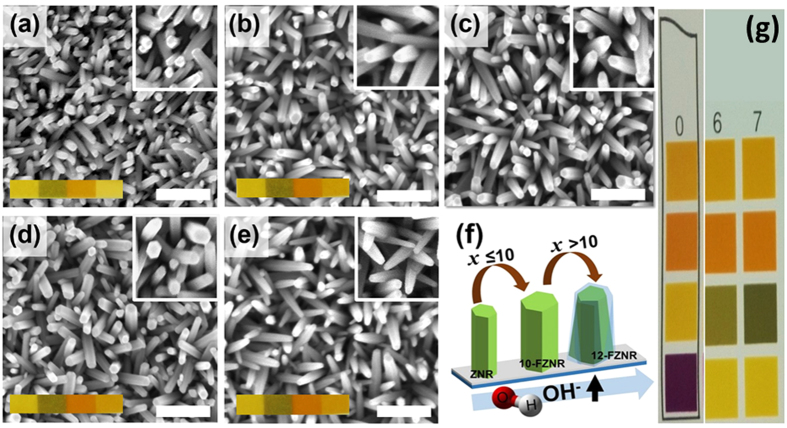
FESEM top-view images of (**a**) ZNR, (**b**) 5-FZNR, (**c**) 8-FZNR, (**d**) 10-FZNR, (**e**) 12-FZNR samples, (**f**) schematic diagram of FZNR growth evolution, and (**g**) standard litmus pH scale. Scale bar represents 100 nm.

Figure 1(a–e) depicts the top-view FESEM images of ZNR and *x*-FZNR on FTO substrate. The as-grown ZNR exhibited an average diameter of (30 ± 2) nm and an effective thickness of (197 ± 6) nm. After F-doping, the average diameter and effective thickness of nanorods were found to increase correspondingly with *x* up to 10 wt%. Specifically, 10-FZNR sample achieved the optimum average diameter and thickness of (38 ± 3) nm and (288 ± 5) nm, respectively (Table S1). Fascinatingly, a morphology evolution was observed when *x* was further increased to 12 wt%, in which the rod-shaped ZNR arrays were transformed into taper-shaped arrays with a much smaller diameter (26 ± 3) nm. Moreover, the average surface density of nanorods was found to increase from 275 (pristine ZNR) to 320 rods/μm^2^ (10-FZNR) before it drastically reduced to 268 rods/μm^2^ (12-FNZR). Primarily, the morphology and kinetic growth rate (both axial and radial) of ZNR is governed by *x*, which directly controls the formation density of F^−^ and hydroxyl ions (OH^−^) during the growth process as well as the pH growth environment, as illustrated in Fig. 1(f). Therefore, the concentration of NH_4_F plays a critical role in dictating the nucleation rate and the crystallographic orientation of FZNR. Herein, the crystal growth process of FZNR can be distinguished into two main parts: (i) surface thermodynamics and (ii) reaction kinetic of nucleation and crystal growth under alkaline condition. The detailed crystal growth mechanism of FZNR and the contemporary ionic reactions under *x* variation were further elucidated in Supplementary Information.

**Figure 2:**
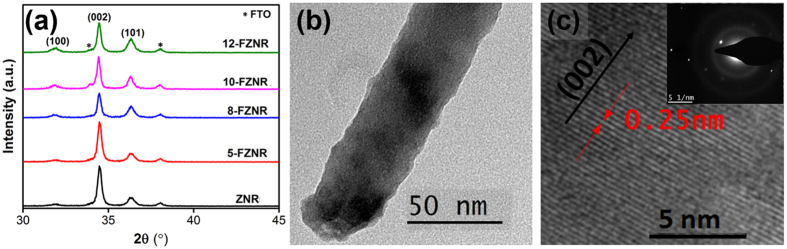
(**a**) X-ray diffraction patterns of ZNR and *x*-FZNR samples, (**b**) HRTEM images and (**c**) magnified HRTEM images followed by inset SAED pattern of 10-FZNR sample.

The microstructural properties of ZNR and *x*-FZNR samples were examined via XRD analysis and the results are presented in Fig. 2(a). All of the XRD patterns displayed four main peaks, namely (100), (002), (101) and (102) which could be indexed to the standard diffraction pattern of a hexagonal wurtzite ZnO structure (JCPDS 36-1451). The dominant (002) peak observed in each spectrum implies that the preferential growth direction of nanorods is along the c-axis orientation. Besides, the negligible shift in 2θ of (002) peak indicates that there is no severe distortion on ZnO lattice because of the relatively similar ionic radius between F^−^ (1.36 Å) and O^2−^ (1.40 Å)^13^. Furthermore, the ratio of (002)/(100) and (002)/(101) of the samples reduced noticeably with increasing *x*, reflecting the inhibited growth of ZNR along the c-axis orientation (Table S2). This phenomenon arises mainly from the presence of superfluous OH^−^ that capped on the highest surface energy (002)_ZnO_ Bragg plane during growth process, leading to a randomly oriented nucleation site^14^,^15^. This finding agrees well with other studies which reported that F^−^ would promote the growth of ZnO crystals along the (101) and (100) plane via occupation of oxygen vacancies (V_o_) in ZnO lattice and therefore, hinder the growth of nanorods along (002) plane^16^,^17^. Meanwhile, the crystallite size of ZnO was evaluated via Debye-Scherrer equation^18^ and it was found to increase from 38 nm (ZNR) to 48 nm (10-FZNR). The larger ZnO crystallite size after doping can be accounted to the expansion of molecular orbital (coulombic repulsion) which arises from the electronegativity difference between Zn and F atoms. When *x* was further increased to 12 wt%, the crystallite size of ZNR reduced. This phenomenon can be ascribed to the presence of superfluous OH^−^ in growth solution that promoted the ionic dissociation and formation of ZnF(OH), of which is detrimental to the growth rate and crystallinity of nanorods. The single crystalline nature of 10-FZNR was confirmed via HRTEM analysis, whereby a continuous lattice fringe (0.26 nm) could be observed from the images, as presented in Fig. 2(b,c). This information suggests that no lattice defect was caused by F^−^ doping. To further verify this claim, SAED diffraction pattern was carried out and the continuous linear diffraction spot in the inset of Fig. 2(c) emphatically reflects the single crystalline crystal diffraction pattern of 10-FZNR. This result correlates well with the aforementioned XRD discussion. An in-depth study concerning the morphology-function relationship of FZNR in the photovoltaic performance HSC device was then conducted.

**Figure 3:**
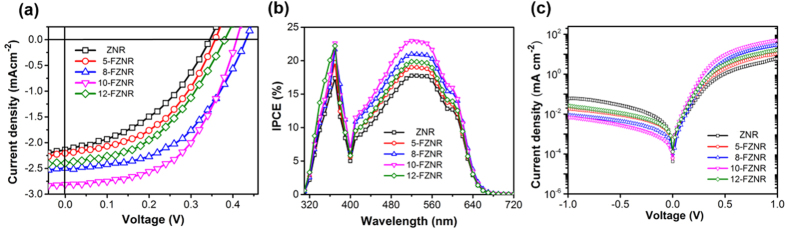
(**a**) J-V characteristic curves under one sun AM1.5 G illumination (100 mW cm^−2^), (**b**) IPCE spectra measured under ambient environment, and (**c**) dark J-V characteristic curves.

**Figure 4:**
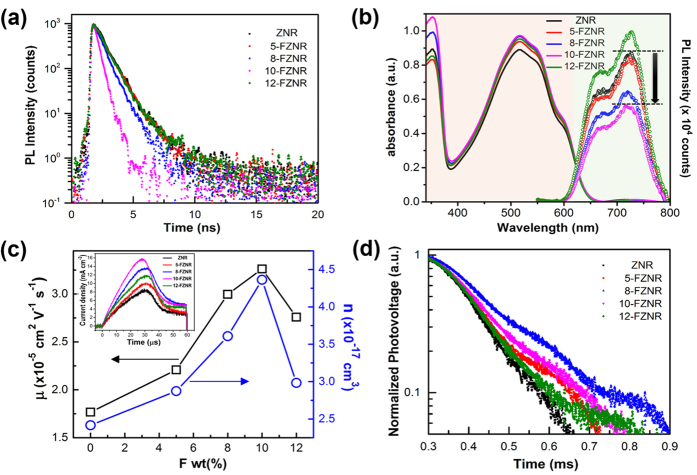
(**a**) TRPL decay curves, (**b**) Combined UV-vis absorption and PL emission spectra for ZNR and *x*-FZNR coated P3HT films (**c**) Extracted carrier mobility and concentration by CELIV; inset shows the typical dark CELIV curves, and (**d**) transient photovoltage decay curves for ZNR and *x*-FZNR device.

Figure 3(a) depicts the current-density (J-V) characteristic of pristine and *x*-FZNR/P3HT devices under an AM1.5 G illumination. As presented in Table 1, the power conversion efficiency (PCE) of pristine ZNR device was recorded at 0.31% with a V_oc_ of 0.34 V, short-circuit current density (J_sc_) of 2.14 mA cm^−2^ and a fill-factor (FF) of 0.42. Interestingly, the cell parameters demonstrated remarkable improvements after F doping, particularly for 10-FZNR device which exhibited a V_oc_ of 0.40 V, J_sc_ of 2.84 mA cm^−2^, and a FF of 0.54, resulting in an overall PCE of 0.61%. However, when *x* > 10 wt%, the photovoltaic response of the device declined rapidly, as evidenced by the lower PCE of 12-FZNR device (0.41%). In conjunction with the J-V measurements, incident photon-to-current conversion efficiency (IPCE) measurements were also conducted and the resulting IPCE spectra are shown in Fig. 3(b). Among all of the devices, 10-FZNR device displayed the highest IPCE throughout both UV and visible region (~24% at 520 nm), which is an appreciable improvement from that of ZNR device (~17% at 520 nm). Specifically, the gradual improvement of IPCE in the UV region of the device can be directly correlated to the increasing effective thickness of the nanorods (Table 1), which explains the relatively higher IPCE exhibited by 10-FZNR and 12-FZNR device in the UV region. On the other hand, a contrastively low IPCE is observed for 12-FZNR device in the visible region, which signifies inferior charge generation and collection in the active layer when *x* > 10 wt%. This phenomenon is mainly caused by the (i) reduced ZnO/P3HT interfacial area resulting from the poor infiltration of P3HT into 12-FZNR (FESEM cross-section images; Fig. S1) and (ii) increase of oxygen defects. Contrarily, the infiltration of P3HT is the optimum for 10-FZNR device and this implies that a larger interfacial area is available for charge separation and collection process. It is acknowledged that the IPCE of a device is closely associated with the photoabsorption ability of the photoactive layer^1^,^2^,^19^. Considering that there is negligible difference in the photoabsorption ability of 10-FZNR and 12-FZNR sample in this region (Fig. 4(b)), the IPCE improvement of 10-FZNR in visible region can be substantiated by the reduced recombination losses across the donor-acceptor interface during photocurrent generation. It is also worth mentioning that the calculated current densities from IPCE curves were relatively consistent with the results from the J–V curves (standard error typically below 5%, Table 2). Apparently, the J_sc_ of the device is closely related to the external quantum efficiency.

Simultaneously, the dark J-V curves also reveal that the charge transport barrier of the device was significantly reduced after fluorine doping, particularly for 10-FZNR device. As seen from Fig. 3(c), the reverse saturation current of 10-FZNR device was considerably suppressed and on the other hand, it displayed higher output currents in forward-bias direction than the rest of the devices. The shunt resistance (R_sh_) of 10-FZNR device increased drastically from 812 Ω cm^2^ (pristine device) to 1587 Ω cm^2^, as shown in Table 2. This finding can be primarily attributed to the densely packed growth of nanorods with larger diameter and higher surface density (Fig. 1(d)) after doping, which effectively reduced the resistance along current shunting paths and suppressed the wrong flow of charge carriers. With increasing *x*, the nanorods array with larger active area and smaller interspacing simultaneously functioned as an effective buffer layer to prevent a direct contact between P3HT and FTO, hence reducing the energy loss in carrier transport and enhancing the electron extraction to FTO^20^. Resultantly, the series resistance (R_s_) of the devices were considerably reduced. From here, it can be comprehended that the relatively higher FF displayed by 10-FZNR device (54%) mainly arises from the high R_sh_ and low R_s_ (ideal circuit: R_sh_ ~ ∞; R_s_ ~ 0). The reported trend of R_s_ and R_sh_ are in good accordance with the FF trend of the device, in which all of them are closely associated with the leakage current, carrier transport and bimolecular recombination of charge carriers in the bulk device^21^,^22^,^23^.

Time resolved photoluminescence (TRPL) measurements were then carried out on all *x*-FZNR/P3HT film samples to elucidate the charge separation and recombination dynamics at ZnO/P3HT interface. The fluorescence decay lifetime (τ) of each sample was estimated by fitting the TRPL semi-log decay curve (Fig. 4(a)) using a bi-exponential function. It is noteworthy that a diluted P3HT layer (18 mg/mL) was coated on the *x*-FZNR samples for TRPL measurements to enable a thorough study on the interfacial charge transfer from P3HT to ZnO with respect to different F-doping concentration^24^. The 472.4 nm picosecond laser source was directly used to excite the P3HT coated *x*-FZNR samples and therefore, the acquired TRPL signals directly reflect the fluorescence decay of P3HT in the active layer. The fluorescence decay of P3HT is governed by the charge separation at the ZnO/P3HT interface^6^,^8^. In this study, the τ of active layer was highly reduced after F-doping (Table S2). Among all of the samples, 10-FZNR/P3HT exhibited the shortest τ (464 ps), which is a phenomenal improvement from that of pristine ZNR sample (656 ps). The exciton decay lifetime obtained from TRPL is at times considered to be highly morphology-dependent and defects related^25^,^26^. Herein, the considerably reduced decay lifetime of 10-FZNR/P3HT can be primarily attributed to the significant increase of interfacial area and oxygen defect passivation by F dopants. As observed from FESEM cross-section images and EDX mapping (Fig. S1), the infiltration of P3HT for 10-FZNR device is the optimum among all of the samples, indicating that larger active interfacial area could be offered for exciton dissociation and charge separation^6^,^27^. Identically, the suppression of defect-induced trapping states in ZnO granted a lower charge transfer barrier at donor-acceptor interface and promoted the separation of bounded electron-hole pairs, leading to a reduction in fluorescence lifetime of the sample^28^.

Simultaneously, steady-state UV-vis and PL measurements were also conducted on *x*-FZNR/P3HT samples and the results were combinatorially depicted in Fig. 4(b). Each UV-vis spectrum consists of two main peaks which located at 354 nm and 520 nm, which arises from the optical absorption of ZNR and P3HT, respectively. As observed from the UV-vis spectra, the optical absorption of nanorods (354 nm) in UV region was remarkably enhanced after doping, which could be ascribed to the increased effective thickness, surface density and average diameter of FZNR. In comparison with the pristine sample, the optical absorption of P3HT for all FZNR samples also improved, albeit to a lesser extent. Concurrently, the normalized optical absorption spectrum plotted in the range of 525–625 nm (Fig. S2) shows no changes in the vibronic peaks of P3HT, implying that there is no improvement in the degree of crystallinity and chain ordering of P3HT. Therefore, the optical absorption improvement of FZNR/P3HT samples in the visible region can be decisively alluded to better infiltration of polymer and reduced oxygen defects following F doping. On another note, the PL emission spectra of FZNR/P3HT samples (especially 10-FZNR) quenched noticeably in comparison with the pristine counterpart. Apparently, the larger surface active area of nanorods with increasing *x* provided larger effective interfacial area for exciton dissociation during photocurrent generation, leading to the enhanced photovoltaic performance of FZNR. The shorter PL decay lifetime and reduced PL emission reported herein clearly suggest that more efficient exciton dissociation and charge separation occurred at ZnO/P3HT interface, a phenomenon predominantly ensued from the suppression of the intrinsic defects in ZnO lattice network via F^−^ incorporation. Nonetheless, when x > 10 wt%, the increase of surface defects, reduced density of nanorods and weaker infiltration of polymer exerted adverse effect on the forward charge transport in the device, leading to the weak photovoltaic performance of 12-FZNR device.

Figure 4(c) presents the charge carrier mobility and concentration of the device extracted from the dark CELIV curves (inset) using a renowned approach^29^. The results show that the carrier mobility and concentration for 10-FZNR device was increased by approximately two fold (n = 3.26 × 10^−5 ^cm^2^ V^−1^ s^−1^; μ = 4.3 × 10^17 ^cm^−3^) as compared to ZNR device (n = 1.77 × 10^−5 ^cm^2^ V^−1^ s^−1^; μ = 2.4 × 10^17 ^cm^−3^). This improvement can be directly correlated to the defect-passivation effect of F dopants which inhibited charge trapping phenomenon at ZnO deep level defect states. The incorporation of F- which involves the annihilation of V_o_ and substitution of lattice oxygen is also capable of delocalizing extra free electrons into the periodic lattice structure of ZNR, thereby contributing to higher free charge carrier concentration under the presence of an external electric field. The passivation of surface –OH groups (charge trapping sites) following F-doping also leads to remarkably higher carrier mobility of the device. The detailed F^−^ incorporation pathway and defect-passivation mechanism will be further elucidated in the latter part of discussion. Apparently, the higher carrier concentration and mobility after F-doping have profoundly contributed to the improvement in J_sc_ of FZNR based device. As *x* was further increased to 12 wt%, the carrier concentration and mobility of the device were simultaneously reduced owing to the inhibited charge transfer. This explained the hindered photovoltaic performance of 12-FZNR device.

Furthermore, the charge transfer dynamics at P3HT/ZnO interface was concisely studied using transient photovoltage (TPV) analysis and the normalized TPV decay curves are depicted in Fig. 4(d). The measured recombination lifetime (τ_rec_) for ZNR device was 84 μs. After F-doping, the τ_rec_ of the device was considerably prolonged and precisely, the optimum τ_rec_ was recorded at 131 μs for 8-FZNR device followed by 122 μs for 10-FZNR device. The result agrees well with the V_oc_ trend of the device, implying that the bimolecular recombination of charge carriers at defect-trapping states (V_o_ and –OH) in ZnO was restricted after the incorporation fluorine. Hindered recombination directly promoted the generation of free charge carriers, which predominantly created a larger energy band offset between the quasi-Fermi levels of holes (HOMO of P3HT) and electrons (CB of ZnO)^23^,^30^,^31^. As a result, the V_oc_ of the device showed corresponding improvement. Nevertheless, the τ_rec_ reduced to 107 μs for 12-FZNR device, in view of the poorer crystallinity of the nanorods and doping-induced defects in ZnO host lattices which act as recombination centers during interfacial charge transfer.

**Figure 5:**
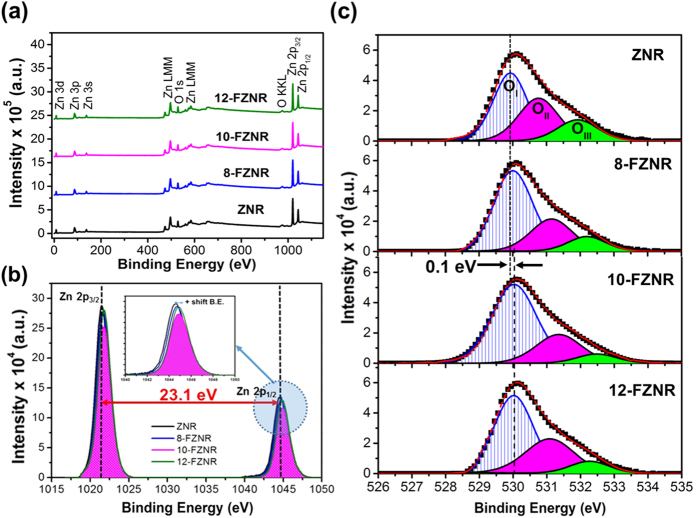
(**a**) Wide**-**scan XPS spectra for ZNR and *x*-FZNR samples, narrow-scan XPS spectra of (**b**) Zn 2p peaks and (**c**) O 1s peak for ZNR and *x*-FZNR samples.

In order to justify the charge extraction improvement in the device, an in-depth study concerning the ionic state energy and chemical composition of ZNR and *x*-FZNR were conducted using XPS analysis. From XPS survey scan (Fig. 5(a)), all of the peaks can be symmetrically ascribed to Zn, O, C and Sn elements with the exception of F. The limited XPS detection of F dopants in *x*-FZNR samples can be explained by its chemical properties: (a) a low molecular weight of 18.99 g mol^−1^ and (b) high electronegativity and chemical reactivity that promotes the formation of ionic and dative covalent bond with surrounding reactive cation such as hydrogen during growth stage. Despite the subtle detection in F 1s region, the presence of F in ZNRs can be alternatively confirmed via the apparent energy shift in other elements.

Basically, the Zn 2p narrow scan of ZNR in Fig. 5(b) consisted of two distinct spectra corresponding to Zn 2p_3/2_ (1021.4 eV) and Zn 2p_1/2_ (1044.5 eV) peak with a spin-orbital splitting (Δmetal) of 23.1 eV, which directly confirmed the +2 oxidation states of Zn atoms^32^. After F-doping, the binding energy (BE) of both Zn 2p peaks gradually shifted upwards and the maximum positive shift (+0.4 eV for Zn 2p_1/2_; +0.5 eV for Zn 2p_3/2_) was observed in 12-FZNR (Fig. 5(b)). This observation implies that the electronic band structure of ZnO was altered after the incorporation of F^−^ into ZnO lattice network. For clarification, the formation of single covalent bond between ZnO and ZnF_x_(OH)_y_ during growth process involves different net charge transfer. Considering that the electronegativity of F^−^ is stronger than O^2−^, the net charge transfer of Zn → F would be comparatively dominant than that of Zn → O. Consequently, the valence electron density between Zn and O was reduced after F-doping, thus leading to the de-shielding of the nucleus in Zn atom and contributed to the upward BE shift of Zn 2p peaks^33^. Nevertheless, the value of Δmetal remained constant for all of the samples, indicating that the subatomic structure and core-level of Zn in ZnO host lattice is independent of F doping concentration.

On top of this, the oxygen-related defects states and chemical stoichiometry ZNR and *x*-FZNR samples were also carefully evaluated by deconvoluting the overlapped O 1s narrow scan spectra (Fig. 5(c)). Each of the deconvoluted spectrum (via Gaussian fitting) consisted of three different regions, denoted as O_I_ (530 eV), O_II_ (531 eV) and O_III_ (532 eV). In particular, O_I_ represents lattice oxygen (Zn-O), O_II_ represents oxygen vacancy (V_O_) and deficiency whereas O_III_ can be assigned to the reactive –OH molecules bounded on ZnO surface^34^,^35^,^36^. To elucidate the interplay between oxygen defects and device performance, the integrated area ratio of O_II_/O_total_ and O_III_/O_total_ were calculated (Table 3). In details, [O_II_/O_total_] indicates the dominancy of V_o_ whereas [O_III_/O_total_] indicates the dominancy of –OH groups in the overall oxygen compartment of ZNR surface. The result reveals that pristine ZNR exhibited the largest oxygen defect fraction among all of the samples, reflecting the typical defect-rich nature of ZNR acquired from hydrothermal synthesis. With increasing *x* up to 10 wt%, a considerable 29% and 59% decrement was recorded in [O_II_/O_total_] and [O_III_/O_total_], respectively for 10-FZNR sample. This finding directly substantiated the novel roles of fluorine as an oxygen defect (V_o_ and –OH) quencher in ZnO lattice. On top of this, a maximum positive B.E shift of +0.1 eV was also witnessed in 12-FZNR sample. This observation could be ascribed to the extra coulombical interactions between the photoemitted electrons and the ion core of oxygen due to the presence of highly electronegative F atoms in ZnO host lattice.

**Figure 6:**
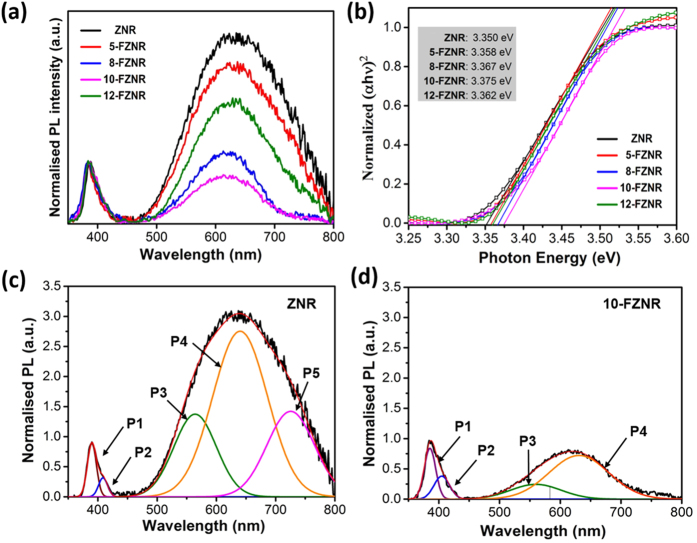
(**a**) Normalized PL spectra, (**b**) Tauc plot of ZNR and *x*-FZNR samples, the deconvoluted PL spectra of (**c**) ZNR and (**d**) 10-FZNR sample.

The additional information concerning the optical and defect properties in ZNR and *x*-FZNR samples were also provided by steady-state PL measurements and the results are shown in Fig. 6. Typically, two main emissions were observed in all PL spectra, namely (a) near band-edge emission (NBE) in UV region (350–400 nm) and (b) deep level emission (DLE) in visible region (400–750 nm). From the normalized PL spectra (with respect to NBE) as displayed in Fig. 6(a), pristine ZNR exhibited the highest DLE among all of the samples and in comparison, 10-FZNR sample possessed the lowest DLE and accordingly, a noticeable improvement in the DLE/NBE ratio. Interestingly, 12-FZNR sample exhibited a higher DLE/NBE ratio than 10-FZNR sample. This phenomenon manifests that the mono-substitutional doping of fluorine into oxygen lattice sites has become increasingly dominant due to the excessive supply of F^−^. This finding correlates well with the former XPS results. The optical energy band gap for each sample was also extracted from the optical absorbance spectra (Fig. S3) using Tauc relation. As observed from Fig. 6(b), the optical band gap of nanorods blue-shifted slightly with increasing *x*, from approximately 3.35 eV (pristine ZNR) to 3.375 eV (10-FZNR). The slight increment in optical band gap can be attributed to Burstein-Moss effect which arises from F-doping^17^,^37^.

In addition, the deconvoluted PL spectra of ZNR and 10-FZNR were presented in Fig. 6(c,d). In NBE region, the overlapped spectra for both samples were successfully fitted into two discrete peaks using Gaussian function, denoted as P1 (VB_Zn_ ↔ CB_Zn_) and P2 (Zn_i_ ↔ VB_Zn_) respectively, whereby Zn_i_ represents Zn interstitials. Interestingly, the NBE peak was hypsochromically shifted with increasing *x*, which is in good correspondence with the blue-shift of optical band gap. Under similar analysis, a significant DLE intensity quench was observed in 10-FZNR sample. In comparison, the P3 (V_o_) and P4 (O_i_ and –OH) regions^38^,^39^,^40^ in 10-FZNR were considerably reduced from that of ZNR. Most notably, the P5 curve (–OH on ZnO surface) completely quenched, reiterating the paramount function of F^−^ in eliminating O-related defects in ZnO^5^,^30^,^40^.

**Figure 7:**
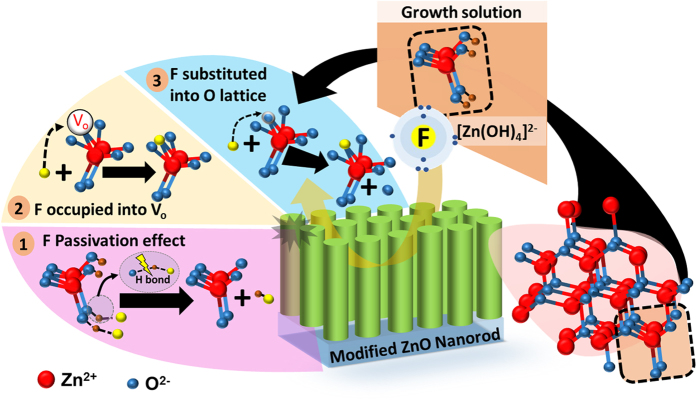
F anion incorporation pathways into ZNR.

Based on a series of compact defect analysis and discussions, a detailed illustration of F^−^ incorporation pathways in ZNR was proposed and illustrated in Fig. 7. The correlations between the lattice point defects in ZnO crystal and its electrical charges properties (carrier mobility and concentration) were established using Kröger–Vink notation. It is commonly known that Schottky defects occurred easily in ZnO lattice during the hydrothermal growth process (equation (1))^3^. It is commonly accepted that the presence of oxygen defects such as V_o_ in ZnO lattice will intrinsically generate more trapping sites during charge transport and hinder the photovoltaic performance of the device. To address the issue, F anion was selected as the oxygen defect quencher because of its peculiar ionic properties (high electronegativity) that could promote ionic reactions on primary growth molecule, Zn(OH)_2_. Essentially, the incorporation of F^−^ into ZnO lattice could be classified into three routes: (1) formation of dative hydrogen bonds (OH-F) with surface hydroxyl groups, (2) occupation of oxygen vacancies and (3) monosubstitution into oxygen lattice sites in ZnO matrix. Intrinsically, the highly reactive F^−^ from NH_4_F dopant source will undergo active interactions with ZnO nuclei and passivate the surface –OH molecules (formed from water hydrolysis and HMT buffer solution), resulting in an acidic growth condition (pH reduced; Fig. 1(g)). Meanwhile, another partition of the F^−^ ions will spontaneously occupy the existing oxygen vacancies or replace the neutral oxygen atom sitting on oxygen lattice site (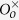) to form a thermodynamically stable neutral F atom (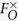) in ZnO lattice^12^,^16^,^41^. It should be noted that oxygen vacancies exist predominately in double ionization state (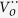) on the surface of hydrothermally grown ZNR because of its low formation energy^9^,^16^. The monosubstitution of oxygen lattice atom by F^−^ and the annihilation of the oxygen vacancies due to the passivation effect of positively charged fluorine ion sitting on oxygen lattice site (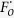) imminently leads to the generation of extra delocalized electron (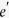), as expressed in eqs (2) and (3)^3^,^16^,^42^.


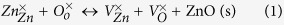



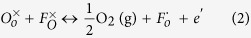



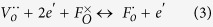


Consequently, the number of delocalized electron in ZNR was increased after the incorporation of F-dopants. To a certain extent, the extra delocalized electrons contributed to the charge extraction by becoming charge carriers themselves. This claim is adequately supported by previous CELIV results which evidenced the increase of extracted charge carriers with increasing fluorine-doping concentration. All of the findings in this study explicitly confirmed the potential of fluorine anion in regulating the material properties and defect states of ZNR, thereby revealing the significance of anion-controlled chemistry in photovoltaic and optoelectronic applications.

## Conclusion

This study has embodied the incorporation mechanism of F^−^ into ZnO host lattice, which primarily occurred via three main routes, i.e. (i) passivation of surface –OH groups via the formation of dative hydrogen bonds (OH-F), (ii) occupation of oxygen vacancies and (iii) monosubstitution into oxygen lattice sites. The incorporation of F-dopants has successfully addressed the defect-rich nature of ZNR and in particular, the oxygen defects (V_o_ and surface –OH groups) were greatly reduced. After F-doping, the photovoltaic performance of HSC was remarkably improved, for instance, the PCE of 10-FZNR device were two-fold higher than that of pristine device. The improvement of the J_sc_ can be mainly ascribed to larger donor-acceptor interfacial area, more efficient exciton dissociation, enhanced charge separation and extraction by ZNR courtesy of lesser defect-induced charge trapping states. Fascinatingly, the incorporation of F-dopants also induced extra delocalized electrons which helped to reduce the energy barrier and improved the V_oc_ of the device. In conclusion, this study has confirmed the effective role of fluorine as an oxygen defect quencher and further yielding the immense potential ZNR in next-generation photovoltaics and optoelectronics application.

## Methods

### Synthesis of ZNR and FZNR

Highly oriented ZNR and fluorine-doped ZNR (FZNR) samples were synthesized on the sol-gel seeded FTO substrate using a conventional hydrothermal approach. In typical procedures, ZNR was prepared from an aqueous precursor solution, which constituted of equimolar (40 mM) hexamethylenetetramine (HMT, Sigma-Aldrich, ≥99.5%) and zinc nitrate hexahydrate (Zn(NO_3_)_2_.6H_2_O, Alfa Aesar, 99.0%). Ammonium fluoride (NH_4_F, Sigma-Aldrich, >99.9%) was used as the fluorine dopant precursor to synthesis FZNR. The structural properties and optical behavior of ZnO were studied as a function of F-doping concentration (*x*), which was fixed as 5, 8, 10 and 12 wt% herein. For device fabrication, a 30 mg/ml of conjugated donor polymer namely poly(3-hexylthiophene-2,5-diyl) (P3HT, Rieke Metals 4002-E) was prepared in chlorobenzene solvent and spin-casted onto ZNR and *x*-FZNR to achieve ~200 nm thick layer. The samples were then post-annealed at 140 °C for 5 minutes to enhance the crystallinity of polymer. Finally, an Ag anode layer with an effective thickness of 120 nm and a defined active area of 0.07 cm^2^ was deposited on the samples using magnetron sputtering technique (50 Watt; Ar: 40 sccm; O_2_: 1 sccm).

### Characterization of ZNR and FZNR

The crystal structures and element purity of the samples were characterized using an X-ray diffractometer, XRD (Bruker AXS D8 Advance). The morphology evolution of each sample was investigated via a field emission scanning electron microscopy, FESEM (Zeiss Merlin Gemini 2) whereas the effective thickness of the samples were measured with a surface profilometer (Veeco Deetak M6). Additionally, the chemical composition and binding energy of the samples were examined by an X-ray photoelectron spectroscopy, (XPS Microprobe, PHI Quantera II), using Al K_α_ as a monochromatic radiation source at room temperature. The optical properties of the samples were studied by a UV-vis-NIR spectrometer (Perkin Elmer Lambda 900) and a steady-state photoluminescence (PL) spectrophotometer (Edinburgh Instruments FLS920) at an excitation wavelength of 300 and 472 nm, respectively. The time-resolved PL (TRPL) spectrum and exciton decay lifetime of *x*-FZNR/P3HT active layers were obtained using a 472.4 nm picosecond laser diode as the excitation source using the same PL set-up.

### HSC Device Characterization

For HSC device analysis, J-V measurements were conducted under dark and light condition using a Keithley 237 SMU under illumination of a solar simulator (100 mW cm^−2^) equipped with an AM 1.5 G filter (Newport 96000). The charge carrier dynamic response of each device was obtained via dark CELIV technique using a voltage pulse of 0.045 V μs^−1^ generated by a function generator (Siglent SDG 1020). The instantaneous charge extraction dynamic response was recorded using a high impedance digital oscilloscope (Siglent 1103CM) with an R_load-series_ of 50 Ω. Transient photovoltage (TPV) measurement was performed on the devices under open-circuit condition with illumination from the solar simulator. A green collimated light emitting diode source (505 nm, repetition rate = 1 kHz, pulse width = 100 μs) was used to generate the perturbation pulse. The photovoltage decay of the devices upon open-circuit voltage (V_oc_) perturbation was recorded by a digital oscilloscope. It should be noted that all of the device fabrication and characterizations reported herein were conducted under standard atmospheric condition (RH of 60–80%).

## Additional Information

**How to cite this article**: Lee, H. B. *et al*. Controlled Defects of Fluorine-incorporated ZnO Nanorods for Photovoltaic Enhancement. *Sci. Rep.* **6**, 32645; doi: 10.1038/srep32645 (2016).

## Supplementary Material

Supplementary Information

## Figures and Tables

**Table 1:** Summarized morphology parameter of ZNR and *x*-FZNR samples.

*x*-FZNR	Average diameter (nm)	Average surface density (μm^−2^)	Effective thickness (nm)
0	30 ± 2	275 ± 12	197 ± 6
5	33 ± 3	296 ± 13	222 ± 8
8	35 ± 2	308 ± 14	251 ± 6
10	38 ± 3	320 ± 16	275 ± 7
12	26 ± 3	268 ± 10	288 ± 5

**Table 2:** Summary of photovoltaic parameters (based on 8 cells) under AM1.5 G illumination (100 mW cm^−2^).

Device (*x*-FZNR)	V_oc_ (V)	J_sc_ (mAcm^−2^)	FF	PCE (%)	R_s_ (Ω cm^2^)	R_sh_ (Ω cm^2^)	Calc. J_sc_ (mAcm^−2^)
0 (ZNR)	0.34 ± 0.02	2.14 ± 0.11	0.42 ± 0.03	0.31 ± 0.02	311	812	2.06
5	0.36 ± 0.02	2.22 ± 0.13	0.46 ± 0.02	0.37 ± 0.02	224	1161	2.19
8	0.42 ± 0.02	2.50 ± 0.18	0.51 ± 0.02	0.54 ± 0.03	91	1465	2.45
10	0.40 ± 0.02	2.84 ± 0.23	0.54 ± 0.02	0.61 ± 0.04	43	1587	2.70
12	0.38 ± 0.03	2.40 ± 0.14	0.45 ± 0.03	0.41 ± 0.03	186	1073	2.28

The calculated J_sc_ values are determined from IPCE results.

**Table 3:** Area under the curve based on the integration peaks of Zn 2p and O 1s for ZNR and x-FZNR samples.

*x*-FZNR	Zn2p		O				[O_I_/O_total_]	[O_II_/O_total_]	[O_III_/O_total_]
	2p_3/2_	2p_1/2_	O_I_	O_II_	O_III_	O_total_			
0 (ZNR)	601819	297015	58433	42634	21258	122325	0.48	0.35	0.17
8	585262	284185	79645	33667	13974	127286	0.63	0.26	0.11
10	552642	260078	88247	32760	9016	130023	0.68	0.25	0.07
12	608405	281339	75611	42341	11914	129866	0.58	0.33	0.09
