# Supplementary material for: Controlled Defects of Fluorine-incorporated ZnO Nanorods for Photovoltaic Enhancement
Source: Sci Rep. 2016 Sep 2;6:32645. doi: 10.1038/srep32645 (PMC5009336; doi:10.1038/srep32645)
Supplement: Supplementary Information [file srep32645-s1.pdf]

Controlled Defects of Fluorine-incorporated ZnO Nanorods for Photovoltaic Enhancement

Hock Beng Lee[1], Riski Titian Ginting[2,*], Sin Tee Tan[1,*], Chun Hui Tan[1], Abdelelah Alshanableh[1], Hind Fadhil Oleiwi[1], Chi Chin Yap[1], Mohd Hafizuddin Hj. Jumali[1] and Muhammad Yahaya[1]

[1]School of Applied Physics, Faculty of Science and Technology, Universiti Kebangsaan Malaysia, 43600 UKM Bangi, Selangor, Malaysia

[2]Department of Flexible and Printable Electronics, Chonbuk National University, Jeonju 561-756, Republic of Korea

*Corresponding author: titianginting@gmail.com; sintee88@gmail.com

Growth Mechanism of FZNR

The ionic formation and reduction of pristine ZNR using hydrothermal approach has been frequently reported, as discussed in equations (1)- (3). Under the anion-controlled growth process, NH_4F was used as the anion precursor source. In initial stage, NH_4F aqueous solution undergoes dissociation and partially hydrolyses to produce highly electronegative F^-, OH^- and NH_3 (equation (4)). As the concentration of NH_4F (x) increases, the formation density of OH^- (basic agent) increases too. Concurrently, H_2O molecules also dissociates into H^+ and OH^-. These ionic species play a key role in controlling the pH of the growth environment. In terms of pH, a HF/ NH_3 (pH buffer) solution was self-generated in between NH_3 and HF, which was formed from the interaction between H+ and F^- (equation (6)). This buffer solution helps to maintain the pH of the growth process and more importantly, it varies with x. From FESEM results, the increased diameter of FZNR with increasing x demonstrated that the additional supply of OH^- from NH_4F has catalyzed the formation of ZnO molecule. However, when x >10 wt%, the OH^- concentration in the solution becomes excessive. The superfluous OH^- in turn accelerates the growth of nanorods and imminently leads to spinodal

decomposition, thus resulting in the taper-shaped arrays in 12-FZNR sample. Simultaneously, the superfluous OH^- also capped on ZnO (0001) polar plane and directly hindered the preferential growth of ZNR along c-axis. The addition of NH_4F into growth solution also triggers other chemical reactions, which are proposed herein along based on their standard enthalpy of formation. Upon the addition of NH_4F, the formation of complex conjugate cation ZnF^+ in the growth solution will become dominant owing to the high electronegativity nature of F^- (equation (7)). ZnF^+ cations are unstable and therefore, they will undergo direct reduction process to form a complex crystal, ZnF(OH) (equation (8)). Subsequently, dual-phase transformations take place in the basic growth solution, specifically (i) ZnF(OH) crystal to $Zn(OH)_2$ and (ii) solid-solid phase transition from $Zn(OH)_2$ to ZnO via lattice dehydration at defect-rich sites (equations (7) and (8)). Fluorine dopants are likely to incorporate into ZnO lattice during these reactions in growth process.

$$Zn^{2+}\,(aq) + 2OH^-\,(aq) \leftrightarrow Zn(OH)_2\,(s)\ \text{(more favorable, } \Delta H_f = -642.9\ \text{kJmol}^{-1}) \quad (1)$$

$$Zn(OH)_2\,(s) + 2OH^-\,(aq) \leftrightarrow [Zn(OH)_4]^{2-}\,(aq) \quad (2)$$

$$[Zn(OH)_4]^{2-}\,(aq) \rightarrow ZnO_{cluster}\,(s) + 2OH^-(aq) + H_2O\,(aq) \quad (3)$$

$$NH_4F\,(s) + H_2O\,(l) \rightarrow NH_3\,(aq) + H_3O^+\,(aq) + F^-\,(aq) \quad (4)$$

$$Zn^{2+}\,(aq) + F^-(aq) \rightarrow ZnF_2\,(s)\ \text{(less favorable, } \Delta H_f = -807.1\ \text{kJmol}^{-1}) \quad (5)$$

$$H^+\,(aq) + F^-(aq) \rightarrow HF\,(l)\ (\Delta H_f = -316.9\ \text{kJmol}^{-1}) \quad (6)$$

$$*Zn^{2+}\,(aq) + F^-(aq) \leftrightarrow ZnF^+\,(aq) \quad (7)$$

$$*ZnF^+\,(aq) + OH^-(aq) \rightarrow ZnF(OH) \quad (8)$$

Table S1. Summarized XRD parameter of pristine ZNR and *x*-FZNR samples.

***x*-FZNR**	**FWHM (^{o})**	**2θ (^{o})**	**Crystallite Size (Å)**	**(002)/(101) Ratio**	**(002)/(100) Ratio**
0 (ZNR)	0.226	34.50	38	3.4	15.2
5	0.212	34.48	41	3.3	13.9
8	0.184	34.45	47	2.0	7.7
10	0.181	34.43	48	2.2	6.3
12	0.197	34.43	43	1.6	5.1

Table S2. Decay lifetime of pristine ZNR/P3HT and *x*-FZNR/P3HT films.

***x*-FZNR/P3HT film**	**Decay Lifetime (ps)**
0 (ZNR)	656
5	644
8	562
10	464
12	673

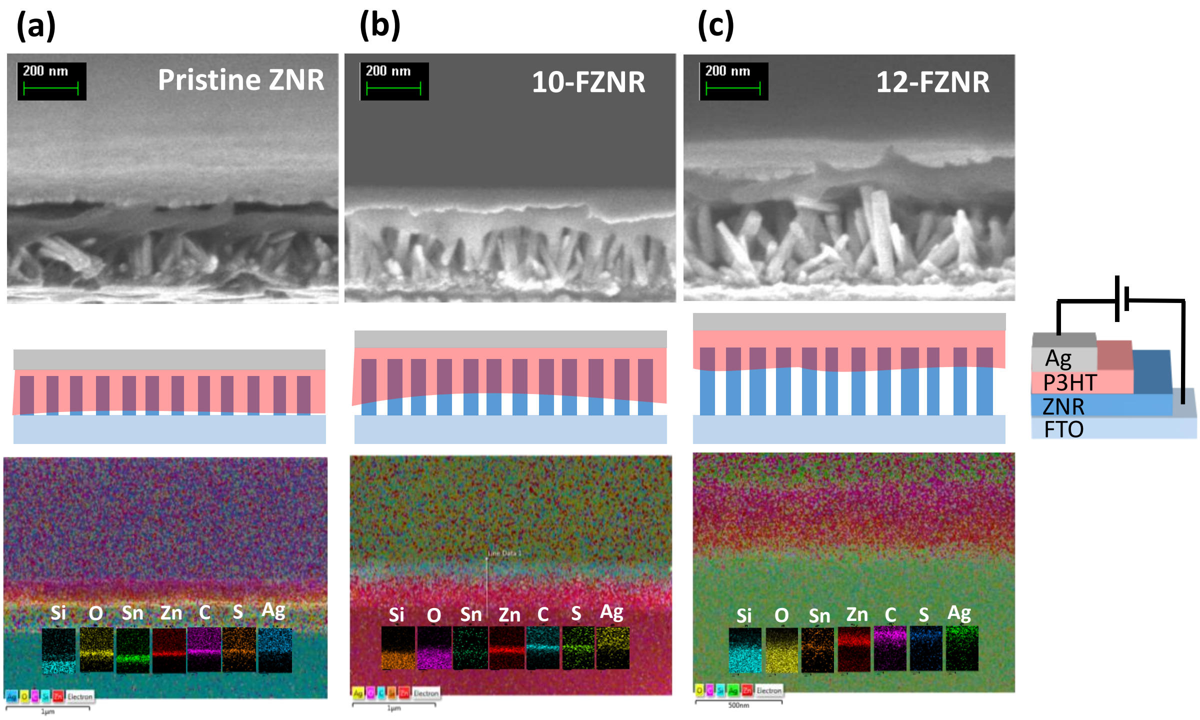

Figure S1. FESEM cross-section images, schematic illustration on P3HT infiltration and corresponding EDX mapping images for (a) pristine (b) 10-FZNR and (c) 12-FZNR device

In Fig. S1, the cross-section FESEM images present the vertically stacked architecture for the as-fabricated FTO/*x*-FZNR/P3HT/Ag device. Basically, it can be observed that the infiltration depth of P3HT reduced correspondingly with F-doping concentration (*x*). As evidenced by 12-FZNR device, the lower infiltration of P3HT has resulted in smaller ZNR/P3HT interfacial area, thereby hindering the interfacial exciton dissociation and charge separation of the device. In comparison, pristine device exhibited larger interfacial contact area. Nonetheless, the direct contact between P3HT and FTO as observed in pristine sample is detrimental to the device performance as it will trigger the wrong flow of charge carriers (holes) via the pinholes in ZnO seed layer to reach FTO cathode, leading to the increase of reverse-bias (leakage) current and low fill factor (FF) of the device. On the other hand, the infiltration of P3HT is the optimum for 10-FZNR sample, thus providing larger active interfacial area during photocurrent generation which directly contributes to better device performance. Additionally, EDX mapping analysis confirmed the existence of elements (Si; O; Sn; Zn; C; S; Ag) in the device and precisely display the distribution of each element. The element map suggests that each layer of the device is constituted of different materials, including (i) substrate and cathode region: Si, Sn and O which mainly originates from the background FTO coated glass substrate, (ii) ETL region: Zn which arises from ZNR layer, (iii) HTL region: C and S which originates from the P3HT layer and (iv) top anode region: Ag which corresponds to the silver contact of the device. The individual element mapping images of C, S, O and Zn in fabricated devices also reflects the infiltration extent of P3HT into ZnO nanorods.

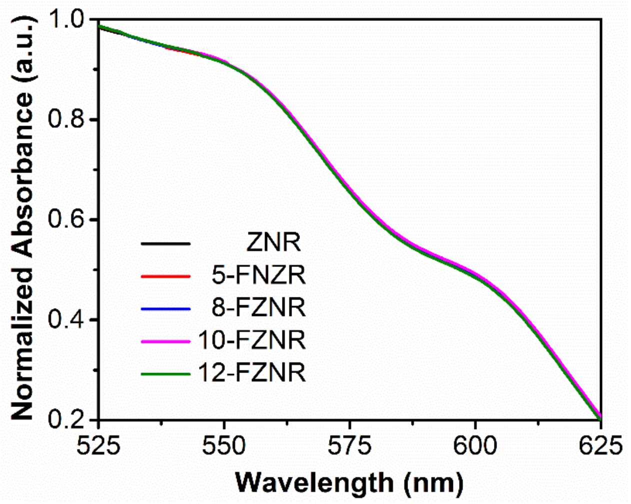

Fig. S2. Normalized UV-vis absorption spectra (525-625) nm for *x*-FZNR/P3HT samples

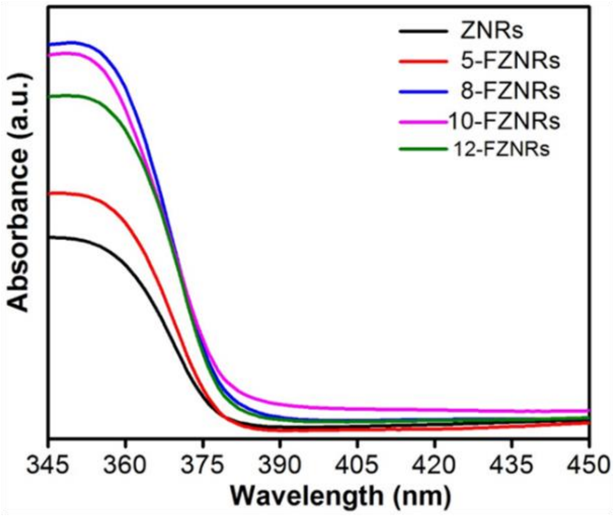

Figure S3. Optical absorbance of *x*-FZNR samples.

The optical properties of ZNR with different F doping concentration as obtained from UV-vis spectrometer (Fig. S2). It is apparent that after F doping, the optical absorption of ZNR improved significantly, which can be correlated with the increased thickness of nanorods. This result was in good agreement with the effective thickness measurement, in which the thickness of nanorod increased from 187 nm (pristine ZNR) to 275 nm (10-FZNR).
